# Exploring objective feature sets in constructing the evolution relationship of animal genome sequences

**DOI:** 10.1186/s12864-023-09747-x

**Published:** 2023-10-24

**Authors:** Xiaolong Li, Hong Li, Zhenhua Yang, Yuan Wu, Mengchuan Zhang

**Affiliations:** 1 Laboratory of Theoretical Biophysics, School of Physical Science and Technology, Inner Mongolia University, Hohhot, 010021 China; 2 School of Economics and Management, Inner Mongolia University of Science and Technology, Baotou, 014010 China

**Keywords:** Genome sequence, 8-mer spectra, Evolution mechanism of genome sequences, Feature set, Evolution relationship

## Abstract

**Background:**

Exploring evolution regularities of genome sequences and constructing more objective species evolution relationships at the genomic level are high-profile topics. Based on the evolution mechanism of genome sequences proposed in our previous research, we found that only the 8-mers containing CG or TA dinucleotides correlate directly with the evolution of genome sequences, and the relative frequency rather than the actual frequency of these 8-mers is more suitable to characterize the evolution of genome sequences.

**Result:**

Therefore, two types of feature sets were obtained, they are the relative frequency sets of CG1 + CG2 8-mers and TA1 + TA2 8-mers. The evolution relationships of mammals and reptiles were constructed by the relative frequency set of CG1 + CG2 8-mers, and two types of evolution relationships of insects were constructed by the relative frequency sets of CG1 + CG2 8-mers and TA1 + TA2 8-mers respectively. Through comparison and analysis, we found that evolution relationships are consistent with the known conclusions. According to the evolution mechanism, we considered that the evolution relationship constructed by CG1 + CG2 8-mers reflects the evolution state of genome sequences in current time, and the evolution relationship constructed by TA1 + TA2 8-mers reflects the evolution state in the early stage.

**Conclusion:**

Our study provides objective feature sets in constructing evolution relationships at the genomic level.

**Supplementary Information:**

The online version contains supplementary material available at 10.1186/s12864-023-09747-x.

## Background

The genome sequence contains information on sequence composition and evolution. The occurrence frequency of k-mers in the genome sequence is considered an ideal feature set for revealing genome information. Based on the non-random feature of k-mer frequency in DNA sequences, functional fragments and functional regions in DNA sequences were analyzed and predicted. Chan and Kibler used 6-mers to predict cis-regulatory motifs [1]. Li and Lin used k-mers (2 ≤ *k* ≤ 9) to predict promoter region [2, 3]. Zhang et al. used k-mers to predict the binding site of locust DNA [4]. Hariharan found that different k-mers were closely related to the diversity of functional fragments [5]. Guo used k-mers to study nucleosome localization [6]. Other studies have mainly focused on probabilistic models of k-mer distribution, rare k-mers, and rich k-mer fragments and their distribution in chromosomes [5, 7–15], and good results have been obtained in searching of some gene regulatory fragments using k-mers [16–19].

Some studies have attempted to study evolution relationships using the non-random features of k-mer usage to overcome the theoretical defects encountered in constructing evolution relationships by employing the sequence alignment method. The study of evolution relationships among species has generally undergone three stages. Initially, evolution relationships were constructed based on single DNA, RNA, or protein sequence; for example, the Fitch used the cytochrome C protein sequence to constructed evolution relationships [20]. Subsequently, many researchers have used the small subunit rRNA sequence [21], elongation factor Tu [22], heat-shock protein (HSP60) gene [23], the largest subunit of RNA polymerase II (RPB1) [24], and aminoacyl-tRNA synthetases [25]. However, owing to the differences in the selected functional sequences, the inconsistency problem of these evolution relationships cannot be solved. In addition, it is not appropriate to use the information of a single segment to represent the evolutionary information of the whole genome sequence. Later, researchers tried to construct evolution relationships by using a set of functional sequences. For instance, Snel used a common gene set [26, 27], Wolf used a conserved cluster of homologous genes [28], and Qi uses all protein-coding sequences or all protein sequences [29–31]. Although this method improves the reliability of evolution relationships, it still cannot resolve the inconsistency problem because there is no uniform standard to select a consistent gene/protein set and to determine the number of selected sets. Furthermore, some genes were not found in some of the analyzed species. The selected sequence set could not represent the whole genome sequence. Some researchers have attempted to construct evolution relationships by aligning whole genome sequences [32, 33]. However, this method is only applicable to mitochondrial genome sequences or very small genome sequences. Currently, there is no method for sequence alignment of larger genomes.

A growing amount of attention has been paid to the information on k-mers frequency in whole genome sequences to construct evolution relationships. Previously, Nussinov explored the compositional heterogeneity of prokaryotic and eukaryotic DNA sequences based on 2-mer frequency [34]. Karlin analyzed and compared the compositional differences in phage, bacterial, and some eukaryotic genome sequences based on k-mers (*k* = 2, 3, 4) frequency [35, 36]. Chapus proposed k-mers frequency difference analysis of whole genome sequences to construct evolution relationships and discussed the optimum range of *k* values [37]. Qi used the k-mer feature set of genome sequences to construct evolution relationships in prokaryotes [29]. However, some insurmountable problems still exist with this method. If all k-mers are selected, the evolution relationships are very poor, particularly in eukaryotes. If the k-mer set is filtered or screened, it is difficult to determine the uniform number of k-mers, and the arbitrariness problem in the selection of the k-mer number cannot be solved. In addition, the selection of k-mer features is also difficult. Evidently, the actual frequency of 8-mers is not a suitable feature. The enormous frequency difference of k-mers seriously undermines the equality of their contributions to evolution. Determining the number and features of k-mers associated with the evolution of genome sequences is currently the most significant challenge.

Several studies have focused on the k-mer spectra of genome sequences and the correlations between k-mer spectra and the evolution of genome sequences. The k-mer spectrum is a specific label for a genome sequence. Chen found that the 6-mer spectra differed for different genome sequences [38]. Subsequently, Chor found that the k-mer spectra of prokaryotes, fungi and some lower animals had single-peak distributions, while the k-mer spectra of higher animals, such as tetrapod mammals, showed a triple-peaked distribution, and speculated that the divergence of k-mer spectra is caused by the interaction between CpG inhibition and G + C content constraint [39].

Our group has studied the spectral features of 48 XY*i* (*i* = 0, 1, 2) 8-mer subsets in 920 eukaryotic and prokaryotic genome sequences. We found that there are the CG- and TA-independent selection modes in the genome sequences, which indicates that CG*i* 8-mers and TA*i* 8-mers play key roles in genome constitution and evolution [40–42]. Finally, we proposed an evolution mechanism of genome sequences, in which the evolution state of a genome sequence is determined by the CG- and TA-independent selections intensities and the mutual inhibition relationship between them. Based on the evolution mechanism, we evaluated the contributions of CG*i* 8-mers and TA*i* 8-mers in the evolution of genome sequences, explored the 8-mer subsets and their features that are directly related to the evolution of genome sequences, and determined a more objective theoretical method to construct evolution relationships. We used this method to construct evolution relationships of mammals, reptiles and insects. Finally, we verified the reliability of our theoretical method using whole-genome sequences.

## Results

### Selection of 8-mer sets related evolution

**Fig. 1:**
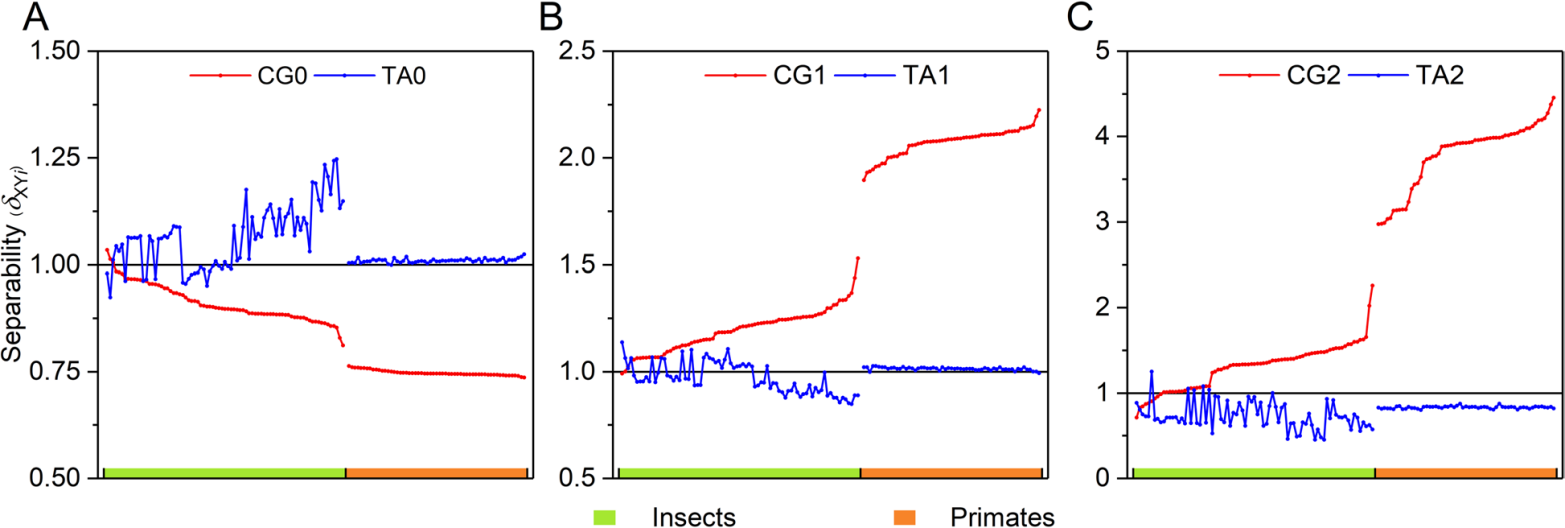
Separability distributions of CG*i* and TA*i* 8-mer spectra in primate and insect genome sequences. **A** The separability distributions of CG0 and TA0 8-mer spectra. **B** The separability distributions of CG1 and TA1 8-mer spectra. **C** The separability distributions of CG2 and TA2 8-mer spectra

The evolution mechanism of genome sequences shows that there are two evolution modes in genome sequences, called the CG- and TA-independent selection modes. The intensity of the CG-independent selection mode can be depicted by the separability values of CG*i* 8-mer spectra (*δ*_CG*i*_), and the intensity of the TA-independent selection mode can be depicted by the separability values of TA*i* 8-mer spectra (*δ*_TA*i*_). Herein, we obtained the intensity distributions of CG- and TA-independent selection for animal genome sequences. The schematic diagram is shown in Fig. 1, the distribution features of invertebrates are represented by insects and the distribution features of vertebrates are represented by primates. We found that: (1) In all of the animals, the intensity of CG1/CG2-independent selection correlated positively with the evolution levels of genome sequences. The intensity of TA1/TA2-independent selection correlated negatively with the evolution levels of genome sequences. The mutual inhibition relationship exists obviously in invertebrate genome sequences. In vertebrate genome sequences, the intensity of TA1/TA2-independent selection falls gradually into the background (*δ* = 1) as the evolution levels of species increase, which implies that the phenomenon of TA-independent selection and the mutual inhibition relationship disappeared gradually [42]. (2) The intensity distributions of the CG0 or TA0 8-mer subset is exactly opposite to those of CG1/CG2 or TA1/TA2 8-mer subsets (Fig. 1). This indicates that the CG0 or TA0 8-mer spectral features are also correlated with the evolution of genome sequences.

Based on the above conclusion, we considered that not all 8-mers correlated directly with the evolution of genome sequences. Hence, determining the directly correlated 8-mer set is a key problem. When analyzing the CG*i* 8-mer spectral distributions in the analyzed animals, we found that CG1 and CG2 8-mer spectra distribute far from the random center and locate at the low-frequency end. The CG0 8-mer spectra distribute around the random center. The distributions of CG1 and CG2 8-mer spectra are more conservative than that of the CG0 8-mer spectra. If an 8-mer spectral distribution is far from the random center and is highly conservative, the occurrence frequency of these 8-mers must be the result of non-random or directed evolution, and these 8-mers must have special biological functions. The results indicated that CG1 and CG2 8-mers correlate directly with the evolution of genome sequences. The same properties were observed for the spectral distributions in the TA*i* 8-mer subsets.

Although the distribution characteristics of CG0 and TA0 8-mers showed a correlation with the evolution of genome sequences, after verification, we found that the correlation is caused by non-random sampling. According to statistical theory, when 8-mers with low frequency are extracted from the total 8-mers population, the average frequency of the remaining 8-mers must increase, and vice versa. This implies that the independent selection intensity must have a negative correlation between CG1/CG2 and CG0 8-mer spectra and between TA1/TA2 and TA0 8-mer spectra. For CG0 or TA0 8-mers, the correlations with the evolution of genome sequences are caused by 8-mers containing CG or TA dinucleotides. In a word, the 8-mers containing CG or TA dinucleotides correlate directly with the evolution of genome sequences and CG0 or TA0 8-mers correlate indirectly with the evolution of genome sequences.

Therefore, we selected CG1 + CG2 8-mers and TA1 + TA2 8-mers as two sets to characterize the evolution state of genome sequences. The number of 8-mers are 24,991 in the two sets which is selected from a total of 65,536 8-mers.

### Selection of evolutionary feature

Determining the feature of the selected 8-mers is another key problem. Generally, the frequency of k-mers was used to characterize the evolution relationship. However, the results were usually unsatisfactory. We considered that the actual frequency of k-mers is not a suitable feature for constructing evolution relationships. Since the spectral distributions of 8-mer subsets are not normal distribution, but more like Poisson distribution, the frequency of more 8-mers is much larger. Using 8-mers with extremely high or extremely low frequency to characterize the evolution of genome sequences is bound to exaggerate or weaken the contribution of these 8-mers. We believed that the suitable feature of each selected 8-mer not only reflect its preference, but also highlight its equality. So, we proposed the relative frequency as the 8-mer feature. The relative frequency is defined as follows: the 8-mers in their subset are arranged in order of their actual frequencies from small to large, and the numerical order is defined as the relative frequency of these 8-mers. The feature not only reflect its preference, but also highlight its equality, and partly eliminates the unequal weight status of the 8-mers in contributing to the evolution of genome sequences. We considered that the number of the obtained 8-mers are objective and have not interfered by man-made, the feature of the relative frequency is more suitable for characterizing the evolution of genome sequences.

### Construction of evolution relationships

#### Evolution relationships of primate genome sequences

**Fig. 2:**
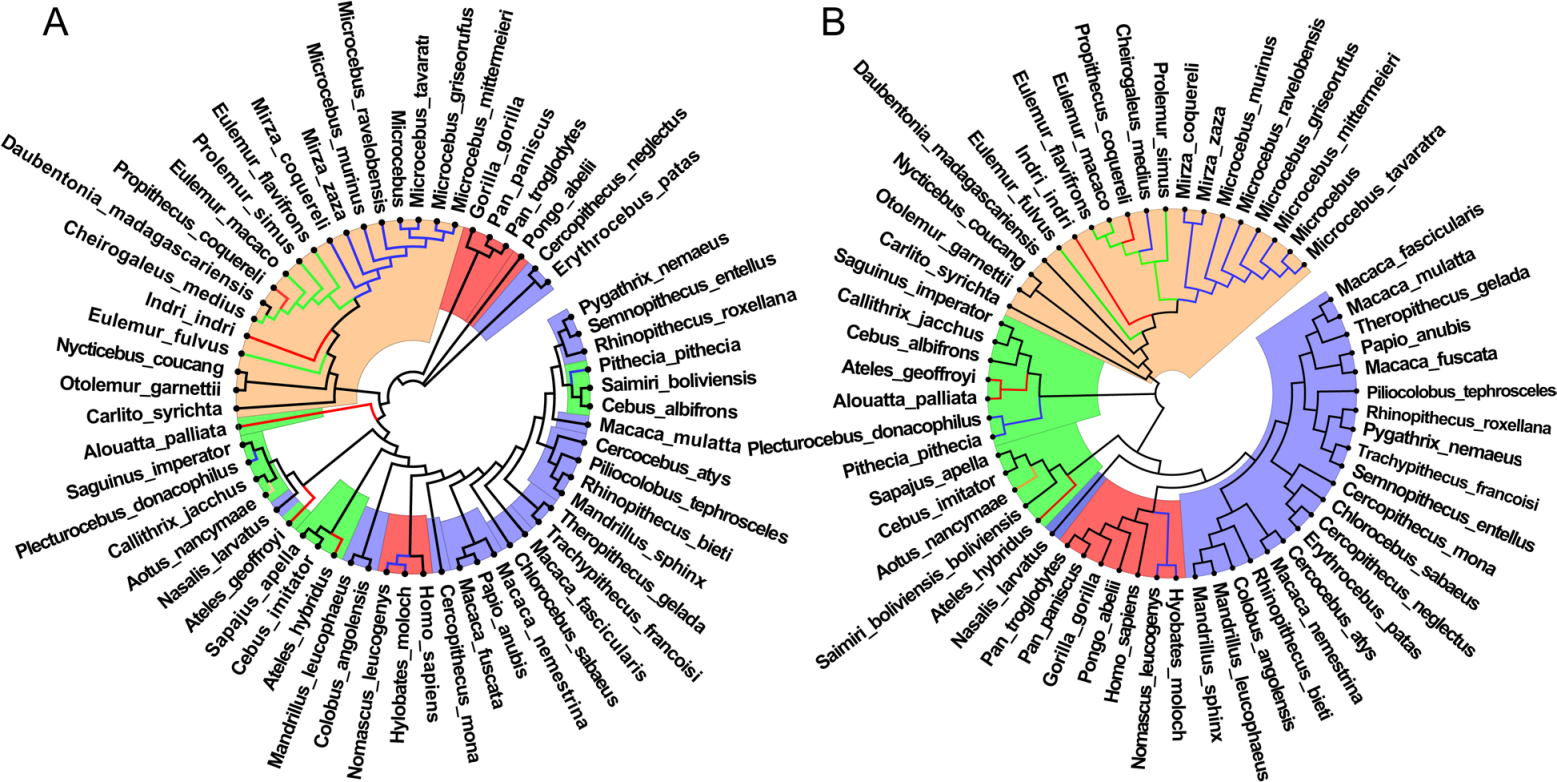
Evolution relationships of primates constructed by total 8-mers. **A** The evolution relationship constructed by the actual frequency set of total 8-mers. **B** The evolution relationship constructed by the relative frequency set of total 8-mers. The blue background represents the old world monkeys, the red background represents the anthropoid, the green background represents the new world monkeys, and the apricot background represents the prosimian

In order to verify the reliability of the feature set we selected, we took the actual frequency and the relative frequency as the features to construct evolution relationships based on total 8-mers. According to ‘Materials and Methods’, the distance matrices are established and the evolution relationships are constructed using Mega7 software between primate genome sequences, the results are shown in Fig. 2.

Based on the actual frequency of total 8-mers, the result showed that only prosimian species are clustered on a main clade, but the other three class species, old world monkeys, anthropoids, new world monkeys, do not form independent clades, they are interlaced with each other (Fig. 2A). The evolution relationship is not consistent obviously with the known conclusion. It means that the actual frequency is not suitable feature. Based on the relative frequency set of total 8-mers, the four class species are clustered basically into four clades (Fig. 2B). But the species in new world monkey (green background) are not clustered into one main clade, and *Nasalis larvatus* is not clustered one clade with old world monkeys (blue background). The results indicated that the relative frequency of 8-mers is suitable feature. Although the evolution relationship is obviously better than that constructed by the actual frequency of total 8-mers, the results also have unacceptable differences compared with the known conclusion. We thought that the differences are caused by the selected total 8-mers.

**Fig. 3:**
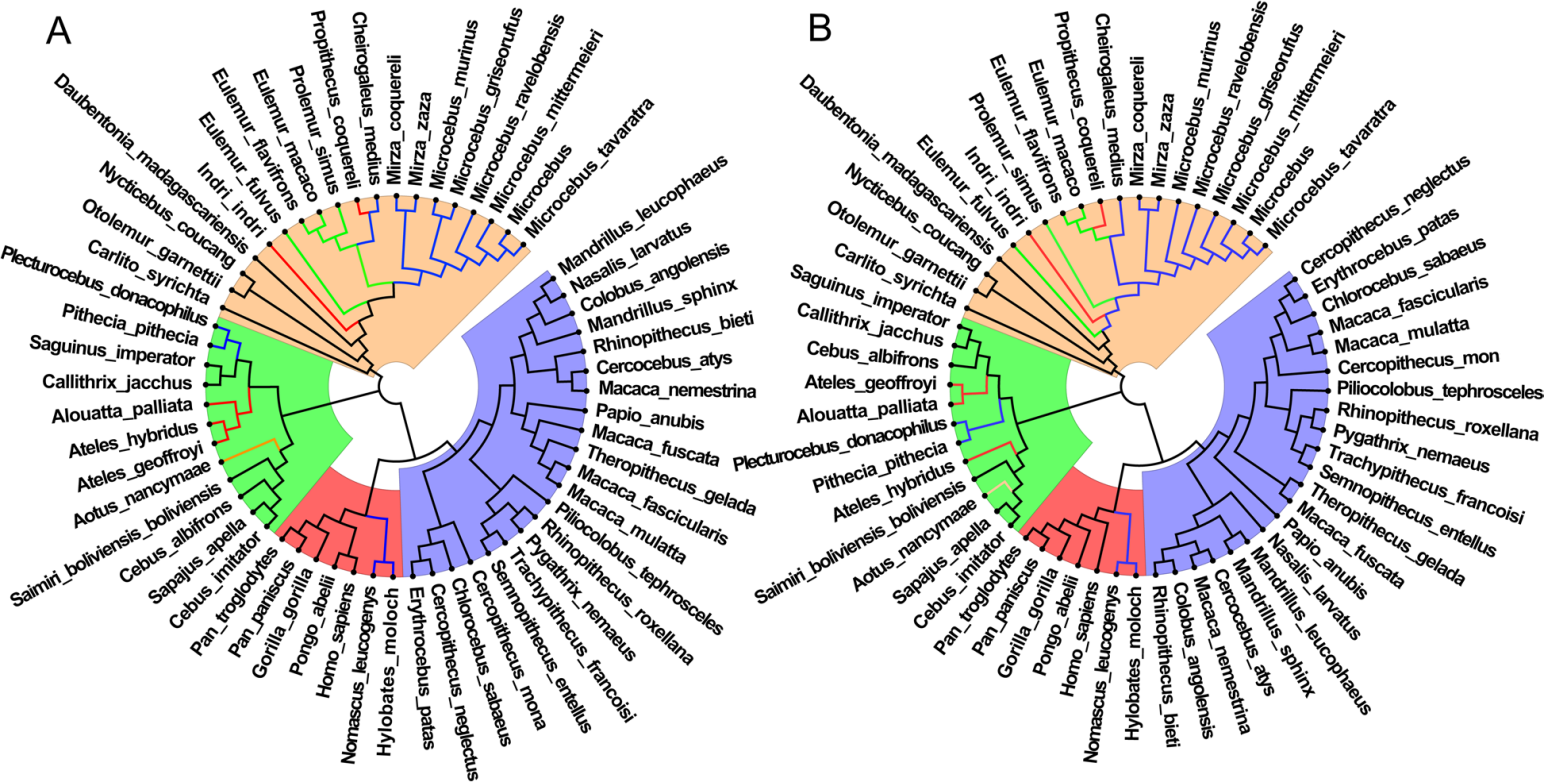
Evolution relationships of primates constructed by CG*i* 8-mers. **A** The evolution relationship constructed by the relative frequency set of CG1 + CG2 8-mers. **B** The evolution relationship constructed by the relative frequency set of CG0 8-mers. The blue background represents the old world monkeys, the red background represents the anthropoid, the green background represents the new world monkeys, and the apricot background represents the prosimian

Because the TA-independent selection mode has disappeared and only the CG-independent selection mode exists in primate genome sequences, we used the features of CG*i* 8-mers to construct the evolution relationships of primates. In order to verify the reliability of our selected feature sets, total 8-mers were divided into two subsets: CG1 + CG2 8-mers (*N*_1_ = 24,991) and CG0 8-mers (*N*_0_ = 40,545). Subsequently, the actual frequency of 8-mers in each set was transformed into relative frequency. Based on the two feature subsets, the evolution relationships of 59 primates were constructed respectively, the results are shown in Fig. 3.

Based on the relative frequency set of CG1 + CG2 8-mers (Fig. 3A), the evolution relationship is consistent with the known conclusions about the evolution relationships of primates [43–46]. Anthropoid, old world monkeys, new world monkeys and prosimian were clearly divided into four main clades. In anthropoid (red background), our results showed that *Homo sapiens* (human) and *Pongo abelii* have the closest evolution relationship, they also formed a clade with *Pan troglodytes*, *Pan paniscus* and *Gorilla gorilla*. *Nomascus leucogenys* and *Hylobates moloch* have the closest evolution relationship. In old world monkeys (blue background), among *Macaca* species, *Macaca mulatta* and *Macaca fascicularis* have the closest evolution relationship and *Macaca fuscata* is adjacent to this clade, but *Macaca nemestrina* is distant from this clade. We also found that *Rhinopithecus roxellana* is closer from *Pygathrix nemaeus*, and is distant from *Rhinopithecus bieti*. In new world monkeys (green background), four *Cebidae* species were clustered into a clade with *Aotus nancymaae*, while the other two *Cebidae* species, *Saguinus imperator* and *Callithrix jacchus*, were clustered into another clade with *Pitheciidae* (blue lines) and *Atelidae* (red lines) species. The evolution relationship is close between the two *Cebidae* species and *Pitheciidae* species. In prosimian (apricot background), *Lemuridae* (green lines) and *Cheirogaleidae* (blue lines) species were clustered into two clades and they are adjacent. We also found that the evolution relationship is distant between *Propithecus coquereli* and *Indre indre*.

Based on the relative frequency set of CG0 8-mers (Fig. 3B), the evolution relationship is basically consistent with that of CG1 + CG2 8-mers. However, there are also some differences between them. But these differences are not obvious in the four main clades. Although the two evolution relationships based on CG1 + CG2 8-mers and CG0 8-mers are similar, according to the evolution mechanism of genome sequences, the 8-mers containing CG dinucleotides correlate directly with the evolution of genome sequences, and CG0 8-mers correlate indirectly with the evolution of the genome sequences. The evolution information contained by CG0 8-mers are caused by non-random sampling. Hence, the features of CG0 8-mers did not provide new evolution information. If we used the CG2 + CG1 + CG0 (total 8-mers) to construct the evolution relationship, it must lead to information redundancy phenomenon, and the information of CG0 8-mers would generate background noise, which would inevitably affect the accuracy of evolution relationship. According to above analysis, we considered that the relative frequency set of CG1 + CG2 8-mers is the best feature parameters to construct evolution relationships.

#### Evolutionary relationships of other vertebrate genome sequences

**Fig. 4:**
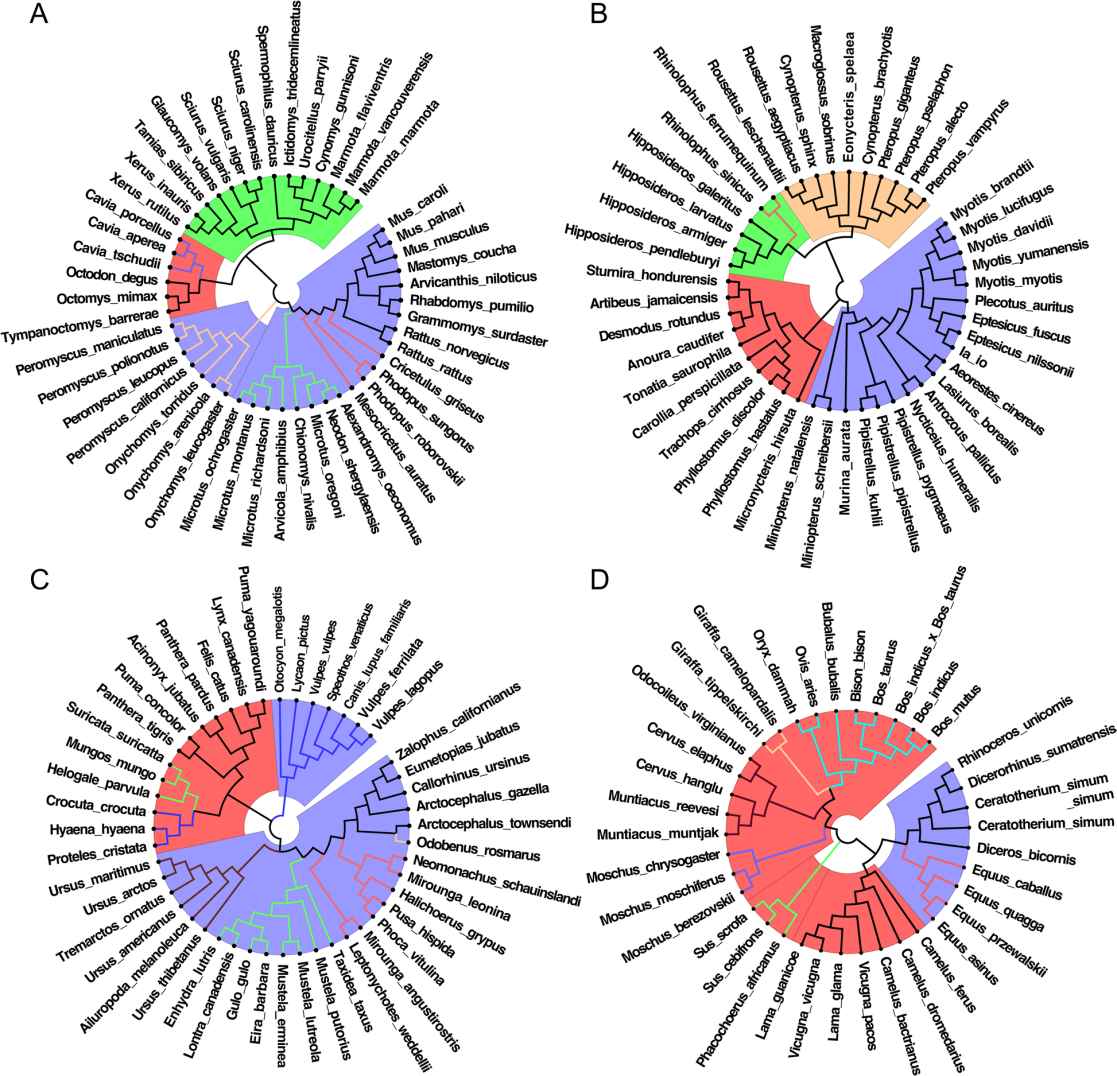
Evolution relationships of other mammal species constructed by the relative frequency set of CG1 + CG2 8-mers. **A** The evolution relationship of *Rodentia* species. The blue background represents *Myomorpha*, the red background represents *Hystricomorpha* and the green background represents *Sciuromorpha*. **B** The evolution relationship of *Chiroptera* species. The blue background represents *Vespertilionidae*, the red background represents *Phyllostomidae*, the green background represents *Rhinolophidae* and the apricot background represents *Pteropodidae*. **C** The evolution relationship of *Carnivora* species. The blue background represents *Caniformia* and the red background represents *Feliformia*. **D** The evolution relationship of *Perissodactyla* and *Artiodactyla* species. The blue background represents *Perissodactyla* and the red background represents *Artiodactyla*

**Fig. 5:**
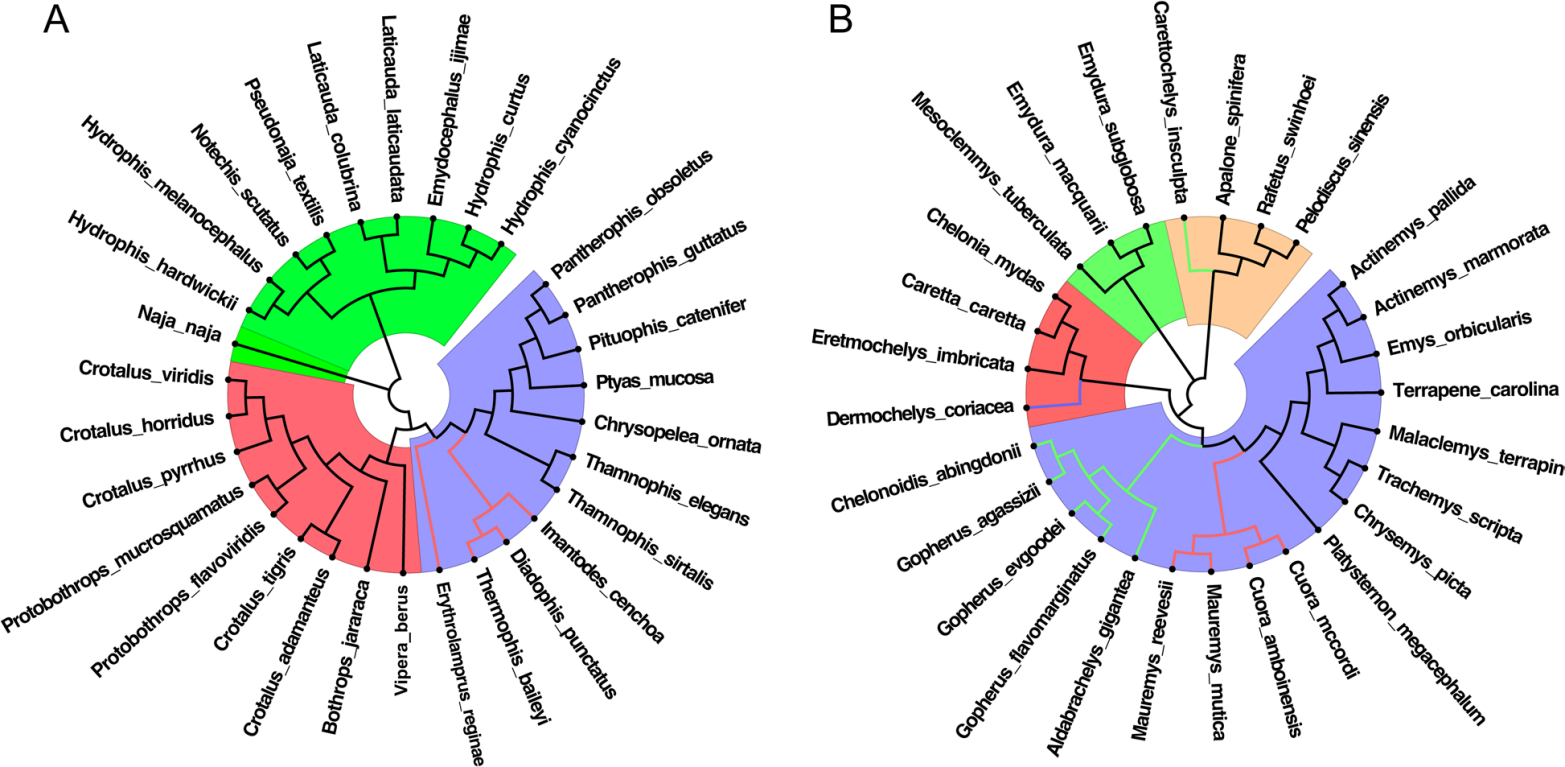
Evolution relationships of reptile species constructed by the relative frequency set of CG1 + CG2 8-mers. **A** The evolution relationship of *Serpentiformes* species. The blue background represents *Colubridae*, the red background represents *Viperidae* and the green background represents *Elapidae*. **B** The evolution relationship of *Tesudines* species. The blue background represents *Testudinoidea*, the red background represents *Chelonioidea*, the green background represents *Chelidae* and the apricot background represents *Trionychia*

Our results shown that the evolution mode of vertebrate genome sequences is dominated by CG-independent selection mode and the TA-independent selection mode disappears basically. In order to verify the universality of the feature set we selected across different species, we constructed evolution relationships based on the relative frequency set of CG1 + CG2 8-mers in other mammals and reptiles. The results are shown in Figs. 4 and 5.

In *Rodentia* species (Fig. 4A), the result showed that *Myomorpha*, *Hystricomorpha* and *Sciuromorpha* are clearly divided into three main clades. Within the main clade of *Myomorpha* (blue background), *Muridae* (black lines) is clearly separated from *Cricetidae*. And *Cricetidae* consists of three single clades, *Cricetinae* (red lines), *Arvicolinae* (green lines) and *Neotominae* (apricot lines). Within the main clade of *Hystricomorpha* (red background), there are two single clades, *Octodontidae* (black lines) and *Caviidae* (blue lines). Within the main clade of *Sciuromorpha* (green background), the only family is *Sciuridae* (black lines). In *Chiroptera* species (Fig. 4B), the result showed that *Vespertilionidae*, *Phyllostomidae*, *Rhinolophidae* and *Pteropodidae* are clearly divided into four main clades. Among them, *Rhinolophidae* (green background) consists of two single clades, *Rhinolophinae* (red lines) and *Hipposiderinae* (black lines). In *Chiroptera* species (Fig. 4C), the result showed that *Otariidae* (black lines) and *Odobenidae* (apricot lines) have the closest evolution relationship, they formed the *Pinnipedia* clade with *Phocidae* (red lines). And *Pinnipedia*, *Mustelidae* (green lines) and *Ursidae* (brown lines) formed the main clade of *Caniformia* (blue background). However, the ingle clade of *Canidae* (blue lines) is not clustered into the main clade of *Caniformia*. Three single clades of *Felidae* (black lines), *Herpestidae* (green lines) and *Hyaenidae* (blue lines) are clustered into a main clade of *Feliformia* (red background). In Fig. 4D, *Perissodactyla* and *Artiodactyla* are clearly divided into two main clades. In *Perissodactyla* (blue background), *Equidae* (red lines) and *Rhinocerotidae* (black lines) are clearly divided into two single clades. In *Artiodactyla* (red background), *Camelidae* (black lines), *Suidae* (green lines), *Moschidae* (blue lines), *Cervidae* (brown lines), *Giraffidae* (apricot lines) and *Bovidae* (cyan lines) all form single clades.

In *Serpentiformes* species (Fig. 5A), the result showed that *Colubridae*, *Viperidae* and *Elapidae* are clearly divided into three main clades. And within *Colubridae* clade (blue background), *Natricinae* (black lines) is clearly separated from *Dipsadinae* (red lines). In *Testudines* species (Fig. 5B), *Testudinoidea*, *Chelonioidea*, *Chelidae* and *Trionychia* are clearly divided into four main clades. *Testudinoidea* (blue background) consists of three single clades, *Emydidae* (black lines), *Geoemydidae* (red lines) and *Testudinidae* (green lines). *Chelonioidea* (red background) consists of two single clades, *Cheloniidae* (black lines) and *Dermochelyidae* (blue lines). *Trionychia* (apricot background) consists of two single clades, *Trionychidae* (black lines) and *Carettochelyidae* (green lines).

For the four kinds of other mammals and two kinds of other vertebrates, the evolution relationships are consistent with the known conclusions [47–55] and the effect of classifications is satisfactory. Therefore, we believed that our method for constructing evolution relationships can be applied to other animal species, and that the feature set is universal across different species.

#### Evolution relationships of insect genome sequences

**Fig. 6:**
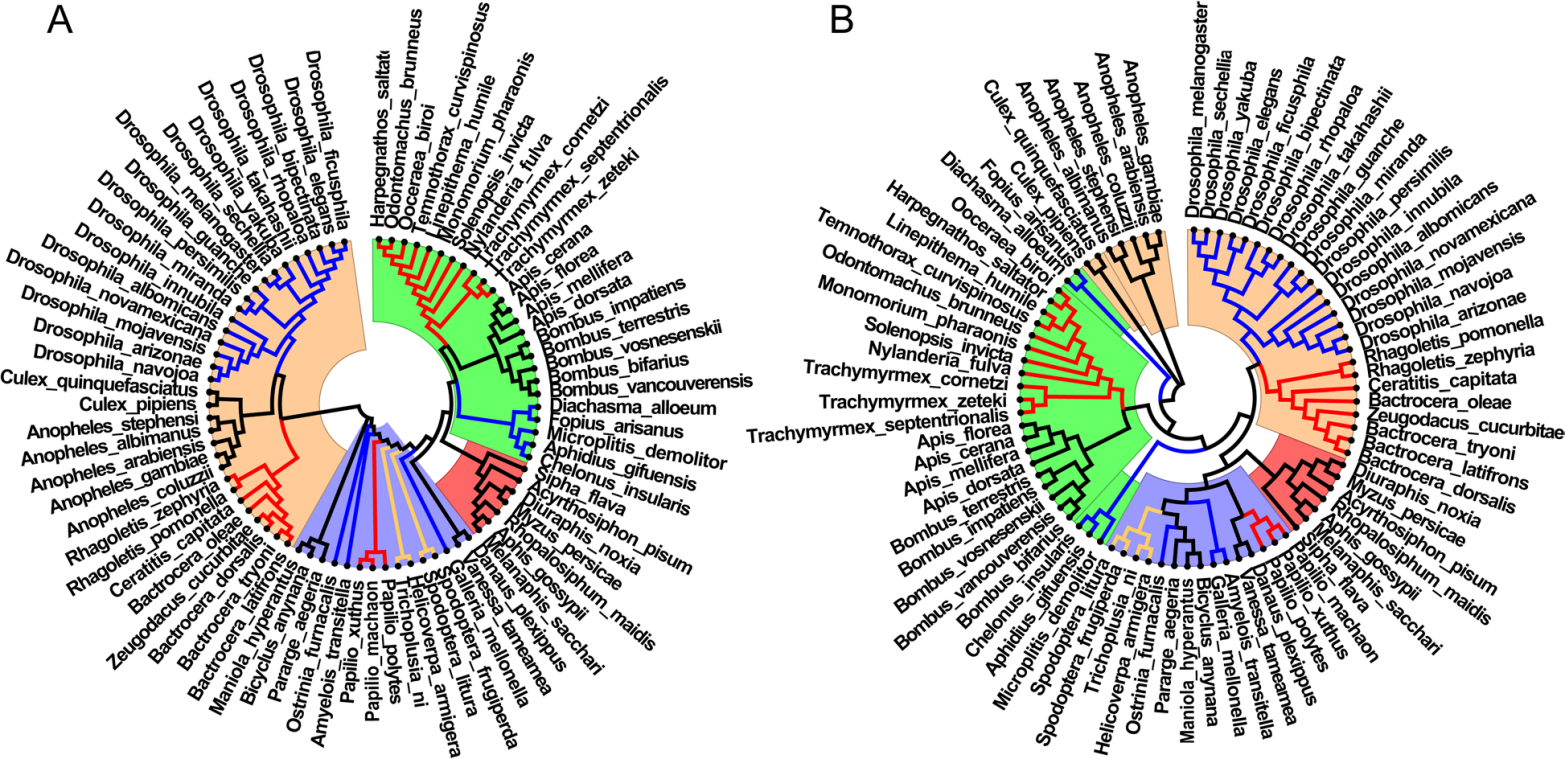
Evolution relationships of insects. **A** The evolution relationship constructed by the relative frequency set of CG1 + CG2 8-mers. **B** The evolution relationship constructed by the relative frequency set of TA1 + TA2 8-mers. The green background represents *Hymenoptera*, the red background represents *Hemiptera*, the blue background represents *Lepidoptrea* and the apricot background represents *Diptera*

In insect genome sequences, both the CG- and TA-independent selection mode exist. Based on the conclusion in primate genome sequences, we are no longer considering the effects of CG0 and TA0 8-mers. The 8-mers were divided into two sets: CG1 + CG2 8-mers and TA1 + TA2 8-mers. The actual frequency of 8-mers is converted to the relative frequency in the two 8-mer sets. According to the method provided, the two evolution relationships of 79 insect genome sequences were obtained respectively, the results are shown in Fig. 6. For convenience, the evolution relationship constructed by the relative frequency set of CG1 + CG2 8-mers is called CG map, and the evolution relationship constructed by the relative frequency set of TA1 + TA2 8-mers is called TA map. The CG map is shown in Fig. 6A and the TA map is shown in Fig. 6B.

According to the evolution mechanism of genome sequences and the analysis on primate genome sequences, we believed that the relative frequency sets of CG1 + CG2 8-mers and TA1 + TA2 8-mers are the best feature sets to construct evolution relationships of genome sequences. Based on the idea, we analyzed the two kinds of evolution relationships of insect genome sequences. In CG map, we found that *Hymenoptera*, *Hemiptera*, *Lepidoptera* and *Diptera* are clearly divided into four main clades. In main clade of *Hymenoptera* (green background), *Formicidae* (red lines), *Braconidae* (blue lines) and *Apidae* (black lines) are clearly divided into three single clades. In main clade of *Diptera* (apricot background), *Drosophilidae* (blue lines), *Culicidae* (black lines) and *Tephritidae* (red lines) are divided into three clades. In main clade of *Hemiptera* (red background), *Aphididae* family is clustered into a single clade. In main clade of *Lepidoptera* (blue background), the cluster relations are scattered. The species in four families are divided into eight clades. In a word, our results are consistent with the known evolution relationships of insects [56–59]. In TA map, we found that the evolution relationship is similar to that of CG map. However, there are some clear differences between them. First, seven species of *Culicidae* are not clustered in the main clade of *Diptera* and they formed a single clade. Second, five species of *Braconidae* are divided into two clades. Third, fifteen species of *Lepidoptera* were clustered into a main clade.

We considered that the differences are not caused by the two selected feature sets, it reflects the information differences between them. Combining previous study [42] and our analysis, the mutual inhibition relationship between CG- and TA-independent selection modes originated from the evolution pressure of an aerobic environment. In bacteria, archaea, fungi, plants and invertebrates, these species have to adopt the mutual inhibition mode to adapt to the aerobic environment. With the increase of evolution levels of animals and plants, the CG-independent selection intensity increases and the TA-independent selection intensity decreases. This indicates that the aerobic environment is the direct cause of the enhancement of CG-independent selection intensity. It means that the CG-independent selection mode is the driving force, and the TA-independent selection mode is changed passively under the mutual inhibition restraints. We considered that the CG 8-mers that represent active evolution must reflect the latest evolution information of genome sequences, and the TA 8-mers that represent passive evolution must contain the legacy evolution information of genome sequences in the early stage. Thus, there should be a time-lag effect between the two feature sets. The information of CG1 + CG2 8-mers characterizes the current evolution state of genome sequences, whereas the information of TA1 + TA2 8-mers characterizes the evolution state in the early stage.

According to our conclusion, CG map characterizes the current evolution state and TA map characterizes the past evolution state of insect genome sequences. Thus, the three differences between CG map and TA map reflect more intensive evolution information.

We can see that seven species of *Culicidae* are not clustered into their main clade of *Diptera* in TA map (Fig. 6B, apricot background and black lines). But they are clustered into their main clade of *Diptera* in CG map. In addition, five species of *Braconidae* are divided into two clades in TA map (Fig. 6B, green background and blue lines), and they clustered into their main clade of *Hymenoptera* in CG map. The results indicated that, in the early stage, the evolutionary environment of the seven species of *Culicidae* differ obviously from the other species of *Diptera*, it causes the evolution separation between them. Similarly, the difference of evolutionary environment in the early stage causes the evolution direction of the five species of *Braconidae* that differ obviously from the other species of *Hymenoptera*. As the evolutionary environment became more and more homogeneous, the evolution direction of these species was similar, then they were clustered into their main clade in current time. In addition, fifteen species are clustered into one main clade of *Lepidoptera* in TA map (Fig. 6B, blue background). But the fifteen species are divided into eight single clades in CG map (Fig. 6A, blue background). It indicated that the evolutionary environment of the fifteen species of *Lepidoptera* was similar and they were clustered in one clade in the early stage. Due to the evolutionary environment has changed, their evolution directions appeared difference. It causes that the cluster relations are scattered in main clade of *Lepidoptera* in current time.

According to the evolution mechanism of genome sequences, we proposed two types of feature sets. The information of CG1 + CG2 8-mers reflects the current evolution state, whereas the information of TA1 + TA2 8-mers reflects the evolution state in the early stage or reflects the legacy information in the early stage. It is interesting that there are two types of feature sets in genome sequences to characterize the evolution state in different periods of time.

## Conclusion and discussion

Our previous study revealed the evolution mechanisms of genome sequences [42]. We analyzed the evolution mechanism further based on mammal, reptile and insect genome sequences. We found that the 8-mers containing CG or TA dinucleotide correlated directly, while the CG0 and TA0 8-mers correlate indirectly with the evolution of genome sequences. Therefore, we selected CG1 + CG2 8-mers and TA1 + TA2 8-mers as the feature sets to characterize the evolution relationships of genome sequences. Considering the equality and the preference of the 8-mers to contribute to the evolution of genome sequences, we proposed the relative frequency as the feature of the 8-mers. Therefore, we obtained two types of feature sets to characterize the evolution relationships. We found that the evolution mode of vertebrate genome sequences is dominated by CG-independent selection mode, the TA-independent selection mode disappears basically. Therefore, we constructed evolution relationships based on the relative frequency set of CG1 + CG2 8-mers in vertebrates, including mammals and reptiles, the results are consistent with the known conclusions. It indicated that the feature sets we select is universal across different species. Because the CG- and TA-independent selection modes are obvious in invertebrate genome sequences, two types of evolution relationships of insects were constructed by the relative frequency sets of both TA1 + TA2 8-mers and CG1 + CG2 8-mers. The two types of evolution relationships are similar. However, there are some differences between them. According to the active and passive evolution relations between the CG- and TA-independent selection modes, we considered that the feature of TA1 + TA2 8-mers reflects the evolution information of genome sequences in the early stages, and the feature of CG1 + CG2 8-mers reflects the evolution information of genome sequences in current time. We conjectured that the differences between the two types of evolution relationships could reflect the variation of evolution environment on the earth, the variation information should be reflected in the composition and structure of genome sequences. The evolution time interval displayed by the two feature sets and the evolution process in the time interval should be researched further.

We proposed a theoretical method for constructing evolution relationships based on the evolution mechanism of genome sequences. Our theoretical method involves two novel ideas. (1) The definite 8-mers were identified. In the case of the 8-mers, two sets of 24,991 8-mers were determined, which correlated directly with the evolution of genome sequences, and 40,545 8-mers that correlated indirectly with the evolution of genome sequences were eliminated from the total 65,536 8-mers. Our selection method is objective and solves the puzzling problem of arbitrariness in k-mers screening. (2) A more reliable evolution feature of 8-mers was proposed. The feature of the relative frequency reflects both the equality and the preference for the 8-mers to contribute to the evolution of genome sequences.

The evolution mechanism of genome sequences has species universality. The selected feature sets can be used to construct the evolution relationships of any species. We will further validate the reliability of the selected feature sets for all species from eukaryotes to prokaryotes. We also hope that researchers will participate in this validation work and establish standardized and universal methods to construct more objective evolution relationships at the genomic level.

## Materials and methods

### Dataset

The whole genome sequences and annotation information for 373 species were obtained from NCBI (https://www.ncbi.nlm.nih.gov/). Sex chromosomes were not included in this dataset. The species genome taxonomy were shown in Table 1, see Supplementary Tables S1, S2 and S3 for detailed information.

**Table 1:** Number of specie genome sequences

Species	Number	Species	Number
*Primates*	59	*Perissodactyla & Artiodactyla*	37
*Rodentia*	48	*Serpentiformes*	30
*Chiroptera*	45	*Tesudines*	28
*Carnivora*	47	*Insecta*	79

### Selection of *k* value in k-mers

For the k-mer spectral analysis, we chose *k* = 8. This choice was based on the following considerations. First, when *k* > 6, the statistical results showed that the distributions of the k-mer spectra of the genome sequences tended to be stable. Second, statistical significance should be ensured for the frequency of k-mer occurrences in the genome sequences. If *k* value is too large, the number of k-mer motifs (*N* = 4^*k*^) is too high, and the frequency of some k-mers is zero, resulting in the loss of statistical information. Beny Chor proposed a formula for minimum *k* value estimation: *k* = 0.7*log*_4_*L*, where *L* is the length of DNA sequence [39]. The *k* ≥ 8 for eukaryotic genome sequences and *k* ≥ 6 for prokaryotic genome sequences. Accordingly, we chose *k* = 8 for our study.

### 8-mer spectrum

For a given genome sequence, the frequency of each 8-mer was obtained using a window of 8 bp and step size of 1 bp. If the number of 8-mers that occurs *i* times is *N*_*i*_, the 8-mer relative motif number (*RMN*) is defined as:

$$RMN=\frac{{N}_{i}}{{4}^{8}}$$

The distribution of *RMN* with 8-mers frequency is called the 8-mer spectrum of the genome sequence.

### Classification method of 8-mers

After obtaining the frequency of each 8-mer in a genome sequence, we classified the 8-mer set into different subsets according to the compositional features of 8-mers. The 8-mers containing zero XY (X, Y = A, T, C, G) dinucleotide were classified into the XY0 subset, those containing one XY dinucleotide were classified into the XY1 subset, and those containing two or more XY dinucleotides were classified into the XY2 subset. Theoretically, there were 4^8^=65,536 8-mers. When X ≠ Y, there were 40,545 8-mers in the XY0, 21,468 8-mer in the XY1, and 3523 8-mer in the XY2. When X = Y, the numbers of 8-mers in the three subsets were 44,631, 14,931 and 5974, respectively. This is known as the XY dinucleotide classification method. Thus, total 8-mers were divided into 16 XY classes. In each XY class, there were three XY 8-mer subsets called XY*i* (*i* = 0,1,2). Finally, we obtained the 8-mer spectra of 48 XY*i* subsets in each genome sequence.

### Separability of 8-mer subset spectrum

The average of the spectral distribution is used to represent its distribution characteristics. In order to eliminate the influence of different genome sizes and show the relative position difference of 8-mer spectra in different subsets, the separability values (*δ*_XY*i*_) were defined.

$${ \delta }_{\text{X}\text{Y}i}=\frac{\stackrel{-}{x}}{{\stackrel{-}{x}}_{\text{X}\text{Y}i}}$$

$\stackrel{-}{x}$ is the average frequency of total 8-mers, called the random center. ${\stackrel{-}{x}}_{\text{X}\text{Y}i}$ is the average frequency of XY*i* 8-mers. *δ*_XYi_ represents the separability for the distribution position of XY*i* 8-mer spectrum relative to the random center. *δ*_XY*i*_ > 1 indicates that XY*i* 8-mer spectrum is located at the low-frequency end and is away from the random center. *δ*_XYi_ = 1 indicates that the location of the XY*i* 8-mer spectrum is the same as that of the random center.

In this definition, the separability value is independent of genome size and the absolute position of the subset spectrum; additionally, this parameter can compare not only the distribution difference of different 8-mer subsets within a genome sequence but also the distribution difference of 8-mer subsets among genome sequences.

### Evolution mechanism of genome sequences

Our previous study analyzed the intrinsic regularity of the 8-mer spectra of 48 XY*i* subsets in 920 genome sequences that include bacteria, archaea, fungi, plants and animals [42]. We found the CG and TA independent selection modes in the genome sequences. The two independent selection modes had five properties. (1) Evolutionary independence: only CG*i* and TA*i* 8-mer spectra formed independent single-peak distributions. (2) Evolutionary separability: the distributions of CG*i* and TA*i* 8-mer spectra show distinct phenomena around the random center. This implies that the separability values were not equal to 1. (3) Evolutionary correlations: the separability values of CG*i* and TA*i* 8-mer spectra correlated closely with the evolution levels of genome sequences in animals and plants. We defined the characteristic quantity of separability as the independent selection intensity. (4) Evolutionary homoplasty: there was a positive correlation between the CG1 and CG2 independent selection intensities and between the TA1 and TA2 independent selection intensities; this indicates that their ways of evolution are similar. (5) There is a mutual inhibition relationship between the CG- and TA-independent selection modes (Fig. 1). Therefore, we proposed an evolution mechanism of genome sequences. The CG- and TA-independent selection intensities and the mutual inhibition relationship between them determine the evolution state of genome sequences.

### Construction of evolution relationship

To construct the evolution relationship, the feature difference of the selected 8-mers was used to construct the distance of genome sequences. The distance (*D*_*ab*_) is defined as:

$${D}_{ab}=\sqrt{\frac{\sum _{i=1}^{N}{({x}_{ai}-{x}_{bi})}^{2}}{N}}$$

where *a* and *b* represent two genome sequences, *x*_*a.i.*_ is the *i*-th 8-mer feature in sequence *a*, and *x*_*bi*_ is the *i*-th 8-mer feature in sequence *b*. *N* is the number of selected 8-mers.

Matrix element *D*_*ab*_ was used to construct the distance matrix for a given genome sequence group. Mega7 (http://www.megasoftware.net) was used to construct evolution relationships by employing the neighbor-joining method.

### Supplementary Information

**Additional file 1:  Supplementary Table S1.** The information of *Mammalia* species and genomes used in this study.

**Additional file 2:  Supplementary Table S2.** The information of *Reptilia* species and genomes used in this study.

**Additional file 3:  Supplementary Table S3.** The information of *Insecta* species and genomes used in this study.

## Data Availability

All genome sequences and the corresponding annotation information were obtained from NCBI (https://www.ncbi.nlm.nih.dov/). See Supplementary Tables S1, S2 and S3 for detailed information.
