# Supplementary material for: Exploring objective feature sets in constructing the evolution relationship of animal genome sequences
Source: BMC Genomics. 2023 Oct 24;24:634. doi: 10.1186/s12864-023-09747-x (PMC10594854; doi:10.1186/s12864-023-09747-x)
Supplement: Supplementary file 1 — Additional file 1: Supplementary Table S1. The information of Mammalia species and genomes used in this study. [file 12864_2023_9747_MOESM1_ESM.docx]

**Supplementary table S1.** The information of *Mammalia* species and genomes used in this study.

| **Name** | **Group** | **Taxonomic Category** | | **Genome**  **Size (Mb)** |
| --- | --- | --- | --- | --- |
|  |  | **Order** | **Family** |  |
| *Cheirogaleus medius* | Prosimian | *Primates* | *Cheirogaleidae* | 2121.89 |
| *Microcebus* | Prosimian | *Primates* | *Cheirogaleidae* | 2499.23 |
| *Microcebus griseorufus* | Prosimian | *Primates* | *Cheirogaleidae* | 2838.33 |
| *Microcebus mittermeieri* | Prosimian | *Primates* | *Cheirogaleidae* | 3012.91 |
| *Microcebus murinus* | Prosimian | *Primates* | *Cheirogaleidae* | 2487.41 |
| *Microcebus ravelobensis* | Prosimian | *Primates* | *Cheirogaleidae* | 2416.21 |
| *Microcebus tavaratra* | Prosimian | *Primates* | *Cheirogaleidae* | 2583.97 |
| *Mirza coquereli* | Prosimian | *Primates* | *Cheirogaleidae* | 2340.87 |
| *Mirza zaza* | Prosimian | *Primates* | *Cheirogaleidae* | 2359.34 |
| *Indri indri* | Prosimian | *Primates* | *Indriidae* | 2701.85 |
| *Propithecus coquereli* | Prosimian | *Primates* | *Indriidae* | 2798.15 |
| *Eulemur flavifrons* | Prosimian | *Primates* | *Lemuridae* | 2115.57 |
| *Eulemur fulvus* | Prosimian | *Primates* | *Lemuridae* | 2748.89 |
| *Eulemur macaco* | Prosimian | *Primates* | *Lemuridae* | 2119.88 |
| *Prolemur simus* | Prosimian | *Primates* | *Lemuridae* | 2411.59 |
| *Daubentonia madagascariensis* | Prosimian | *Primates* | *Daubentoniidae* | 2498.42 |
| *Otolemur garnettii* | Prosimian | *Primates* | *Galagidae* | 2519.72 |
| *Nycticebus coucang* | Prosimian | *Primates* | *Lorisidae* | 3294.56 |
| *Carlito syrichta* | Prosimian | *Primates* | *Tarsiidae* | 3453.86 |
| *Gorilla gorilla* | Anthropoid | *Primates* | *Hominidae* | 3044.87 |
| *Homo sapiens* | Anthropoid | *Primates* | *Hominidae* | 3298.43 |
| *Pan paniscus* | Anthropoid | *Primates* | *Hominidae* | 3051.90 |
| *Pan troglodytes* | Anthropoid | *Primates* | *Hominidae* | 3050.40 |
| *Pongo abelii* | Anthropoid | *Primates* | *Hominidae* | 3065.05 |
| *Hylobates moloch* | Anthropoid | *Primates* | *Hylobatidae* | 2848.33 |
| *Nomascus leucogenys* | Anthropoid | *Primates* | *Hylobatidae* | 2843.98 |
| *Cercocebus atys* | Old world monkeys | *Primates* | *Cercopithecidae* | 2848.25 |
| *Cercopithecus mona* | Old world monkeys | *Primates* | *Cercopithecidae* | 2902.80 |
| *Cercopithecus neglectus* | Old world monkeys | *Primates* | *Cercopithecidae* | 3278.86 |
| *Chlorocebus sabaeus* | Old world monkeys | *Primates* | *Cercopithecidae* | 2937.83 |
| *Colobus angolensis* | Old world monkeys | *Primates* | *Cercopithecidae* | 2970.12 |
| *Erythrocebus patas* | Old world monkeys | *Primates* | *Cercopithecidae* | 3490.49 |
| *Macaca fascicularis* | Old world monkeys | *Primates* | *Cercopithecidae* | 2854.88 |
| *Macaca fuscata* | Old world monkeys | *Primates* | *Cercopithecidae* | 2930.71 |
| *Macaca mulatta* | Old world monkeys | *Primates* | *Cercopithecidae* | 2971.33 |
| *Macaca nemestrina* | Old world monkeys | *Primates* | *Cercopithecidae* | 2948.70 |
| *Mandrillus leucophaeus* | Old world monkeys | *Primates* | *Cercopithecidae* | 3061.99 |
| *Mandrillus sphinx* | Old world monkeys | *Primates* | *Cercopithecidae* | 2860.58 |
| *Nasalis larvatus* | Old world monkeys | *Primates* | *Cercopithecidae* | 3011.97 |
| *Papio anubis* | Old world monkeys | *Primates* | *Cercopithecidae* | 2869.82 |
| *Piliocolobus tephrosceles* | Old world monkeys | *Primates* | *Cercopithecidae* | 2997.80 |
| *Pygathrix nemaeus* | Old world monkeys | *Primates* | *Cercopithecidae* | 3217.15 |
| *Rhinopithecus bieti* | Old world monkeys | *Primates* | *Cercopithecidae* | 2975.79 |
| *Rhinopithecus roxellana* | Old world monkeys | *Primates* | *Cercopithecidae* | 3038.48 |
| *Semnopithecus entellus* | Old world monkeys | *Primates* | *Cercopithecidae* | 3033.04 |
| *Theropithecus gelada* | Old world monkeys | *Primates* | *Cercopithecidae* | 2889.63 |
| *Trachypithecus francoisi* | Old world monkeys | *Primates* | *Cercopithecidae* | 2875.57 |
| *Aotus nancymaae* | New world monkeys | *Primates* | *Aotidae* | 2861.68 |
| *Alouatta palliata* | New world monkeys | *Primates* | *Atelidae* | 3033.62 |
| *Ateles geoffroyi* | New world monkeys | *Primates* | *Atelidae* | 2897.03 |
| *Ateles hybridus* | New world monkeys | *Primates* | *Atelidae* | 2643.27 |
| *Callithrix jacchus* | New world monkeys | *Primates* | *Cebidae* | 2897.82 |
| *Cebus albifrons* | New world monkeys | *Primates* | *Cebidae* | 3058.67 |
| *Cebus imitator* | New world monkeys | *Primates* | *Cebidae* | 2717.70 |
| *Saguinus imperator* | New world monkeys | *Primates* | *Cebidae* | 3493.72 |
| *Saimiri boliviensis boliviensis* | New world monkeys | *Primates* | *Cebidae* | 2651.40 |
| *Sapajus apella* | New world monkeys | *Primates* | *Cebidae* | 2729.20 |
| *Pithecia pithecia* | New world monkeys | *Primates* | *Pitheciidae* | 2933.56 |
| *Plecturocebus donacophilus* | New world monkeys | *Primates* | *Pitheciidae* | 2959.76 |
| *Octodon degus* | × | *Rodentia; Hystricomorpha* | *Octodontidae* | 2995.89 |
| *Octomys mimax* | × | *Rodentia; Hystricomorpha* | *Octodontidae* | 2591.29 |
| *Tympanoctomys barrerae* | × | *Rodentia; Hystricomorpha* | *Octodontidae* | 2651.63 |
| *Cavia aperea* | × | *Rodentia; Hystricomorpha* | *Caviidae* | 2716.40 |
| *Cavia porcellus* | × | *Rodentia; Hystricomorpha* | *Caviidae* | 2723.22 |
| *Cavia tschudii* | × | *Rodentia; Hystricomorpha* | *Caviidae* | 3077.35 |
| *Cricetulus griseus* | × | *Rodentia; Myomorpha* | *Muroidea; Cricetidae; Cricetinae* | 2399.79 |
| *Mesocricetus auratus* | × | *Rodentia; Myomorpha* | *Muroidea; Cricetidae; Cricetinae* | 2457.08 |
| *Phodopus roborovskii* | × | *Rodentia; Myomorpha* | *Muroidea; Cricetidae; Cricetinae* | 2384.24 |
| *Phodopus sungorus* | × | *Rodentia; Myomorpha* | *Muroidea; Cricetidae; Cricetinae* | 2119.20 |
| *Onychomys arenicola* | × | *Rodentia; Myomorpha* | *Muroidea; Cricetidae; Neotominae* | 2871.85 |
| *Onychomys leucogaster* | × | *Rodentia; Myomorpha* | *Muroidea; Cricetidae; Neotominae* | 3152.99 |
| *Onychomys torridus* | × | *Rodentia; Myomorpha* | *Muroidea; Cricetidae; Neotominae* | 2468.39 |
| *Peromyscus californicus* | × | *Rodentia; Myomorpha* | *Muroidea; Cricetidae; Neotominae* | 2467.92 |
| *Peromyscus leucopus* | × | *Rodentia; Myomorpha* | *Muroidea; Cricetidae; Neotominae* | 2475.18 |
| *Peromyscus maniculatus* | × | *Rodentia; Myomorpha* | *Muroidea; Cricetidae; Neotominae* | 2512.44 |
| *Peromyscus polionotus* | × | *Rodentia; Myomorpha* | *Muroidea; Cricetidae; Neotominae* | 2645.11 |
| *Alexandromys oeconomus* | × | *Rodentia; Myomorpha* | *Muroidea; Cricetidae; Arvicolinae* | 2306.55 |
| *Arvicola amphibius* | × | *Rodentia; Myomorpha* | *Muroidea; Cricetidae; Arvicolinae* | 2297.77 |
| *Chionomys nivalis* | × | *Rodentia; Myomorpha* | *Muroidea; Cricetidae; Arvicolinae* | 2393.39 |
| *Microtus montanus* | × | *Rodentia; Myomorpha* | *Muroidea; Cricetidae; Arvicolinae* | 2335.50 |
| *Microtus ochrogaster* | × | *Rodentia; Myomorpha* | *Muroidea; Cricetidae; Arvicolinae* | 2287.34 |
| *Microtus oregoni* | × | *Rodentia; Myomorpha* | *Muroidea; Cricetidae; Arvicolinae* | 3017.11 |
| *Microtus richardsoni* | × | *Rodentia; Myomorpha* | *Muroidea; Cricetidae; Arvicolinae* | 2266.59 |
| *Neodon shergylaensis* | × | *Rodentia; Myomorpha* | *Muroidea; Cricetidae; Arvicolinae* | 2252.56 |
| *Arvicanthis niloticus* | × | *Rodentia; Myomorpha* | *Muroidea; Muridae; Murinae* | 2496.76 |
| *Grammomys surdaster* | × | *Rodentia; Myomorpha* | *Muroidea; Muridae; Murinae* | 2412.66 |
| *Mastomys coucha* | × | *Rodentia; Myomorpha* | *Muroidea; Muridae; Murinae* | 2507.18 |
| *Mus caroli* | × | *Rodentia; Myomorpha* | *Muroidea; Muridae; Murinae* | 2553.13 |
| *Mus musculus* | × | *Rodentia; Myomorpha* | *Muroidea; Muridae; Murinae* | 2728.22 |
| *Mus pahari* | × | *Rodentia; Myomorpha* | *Muroidea; Muridae; Murinae* | 2475.01 |
| *Rattus norvegicus* | × | *Rodentia; Myomorpha* | *Muroidea; Muridae; Murinae* | 2647.92 |
| *Rattus rattus* | × | *Rodentia; Myomorpha* | *Muroidea; Muridae; Murinae* | 2382.79 |
| *Rhabdomys pumilio* | × | *Rodentia; Myomorpha* | *Muroidea; Muridae; Murinae* | 2326.17 |
| *Cynomys gunnisoni* | × | *Rodentia; Sciuromorpha* | *Sciuridae* | 2674.37 |
| *Marmota flaviventris* | × | *Rodentia; Sciuromorpha* | *Sciuridae* | 2412.66 |
| *Marmota marmota* | × | *Rodentia; Sciuromorpha* | *Sciuridae* | 2506.85 |
| *Marmota vancouverensis* | × | *Rodentia; Sciuromorpha* | *Sciuridae* | 2504.04 |
| *Spermophilus dauricus* | × | *Rodentia; Sciuromorpha* | *Sciuridae* | 3106.27 |
| *Tamias sibiricus* | × | *Rodentia; Sciuromorpha* | *Sciuridae* | 2643.75 |
| *Urocitellus parryii* | × | *Rodentia; Sciuromorpha* | *Sciuridae* | 2520.51 |
| *Xerus inauris* | × | *Rodentia; Sciuromorpha* | *Sciuridae* | 2601.42 |
| *Xerus rutilus* | × | *Rodentia; Sciuromorpha* | *Sciuridae* | 2750.76 |
| *Glaucomys volans* | × | *Rodentia; Sciuromorpha* | *Sciuridae* | 2582.20 |
| *Ictidomys tridecemlineatus* | × | *Rodentia; Sciuromorpha* | *Sciuridae* | 2478.97 |
| *Sciurus carolinensis* | × | *Rodentia; Sciuromorpha* | *Sciuridae* | 2815.40 |
| *Sciurus niger* | × | *Rodentia; Sciuromorpha* | *Sciuridae* | 2985.24 |
| *Sciurus vulgaris* | × | *Rodentia; Sciuromorpha* | *Sciuridae* | 2878.61 |
| *Aeorestes cinereus* | × | *Chiroptera* | *Vespertilionidae* | 2149.06 |
| *Antrozous pallidus* | × | *Chiroptera* | *Vespertilionidae* | 2126.46 |
| *Eptesicus nilssonii* | × | *Chiroptera* | *Vespertilionidae* | 2064.12 |
| *Eptesicus fuscus* | × | *Chiroptera* | *Vespertilionidae* | 2008.06 |
| *Ia io* | × | *Chiroptera* | *Vespertilionidae* | 2099.75 |
| *Lasiurus borealis* | × | *Chiroptera* | *Vespertilionidae* | 2857.59 |
| *Miniopterus schreibersii* | × | *Chiroptera* | *Vespertilionidae* | 1775.85 |
| *Miniopterus natalensis* | × | *Chiroptera* | *Vespertilionidae* | 1803.10 |
| *Murina aurata* | × | *Chiroptera* | *Vespertilionidae* | 2331.55 |
| *Myotis yumanensis* | × | *Chiroptera* | *Vespertilionidae* | 2050.52 |
| *Myotis brandtii* | × | *Chiroptera* | *Vespertilionidae* | 2107.24 |
| *Myotis davidii* | × | *Chiroptera* | *Vespertilionidae* | 2059.80 |
| *Myotis lucifugus* | × | *Chiroptera* | *Vespertilionidae* | 2034.58 |
| *Myotis myotis* | × | *Chiroptera* | *Vespertilionidae* | 2002.80 |
| *Nycticeius humeralis* | × | *Chiroptera* | *Vespertilionidae* | 2779.57 |
| *Pipistrellus pipistrellus* | × | *Chiroptera* | *Vespertilionidae* | 1763.44 |
| *Pipistrellus pygmaeus* | × | *Chiroptera* | *Vespertilionidae* | 1895.13 |
| *Pipistrellus kuhlii* | × | *Chiroptera* | *Vespertilionidae* | 1775.69 |
| *Plecotus auritus* | × | *Chiroptera* | *Vespertilionidae* | 2128.73 |
| *Anoura caudifer* | × | *Chiroptera* | *Phyllostomidae* | 2206.59 |
| *Artibeus jamaicensis* | × | *Chiroptera* | *Phyllostomidae* | 2207.62 |
| *Carollia perspicillata* | × | *Chiroptera* | *Phyllostomidae* | 2689.41 |
| *Desmodus rotundus* | × | *Chiroptera* | *Phyllostomidae* | 2063.54 |
| *Micronycteris hirsuta* | × | *Chiroptera* | *Phyllostomidae* | 2314.65 |
| *Phyllostomus hastatus* | × | *Chiroptera* | *Phyllostomidae* | 2089.22 |
| *Phyllostomus discolor* | × | *Chiroptera* | *Phyllostomidae* | 2108.83 |
| *Sturnira hondurensis* | × | *Chiroptera* | *Phyllostomidae* | 2096.62 |
| *Tonatia saurophila* | × | *Chiroptera* | *Phyllostomidae* | 2105.89 |
| *Trachops cirrhosus* | × | *Chiroptera* | *Phyllostomidae* | 2179.54 |
| *Hipposideros galeritus* | × | *Chiroptera* | *Hipposideridae* | 2440.75 |
| *Hipposideros larvatus* | × | *Chiroptera* | *Hipposideridae* | 2291.76 |
| *Hipposideros pendleburyi* | × | *Chiroptera* | *Hipposideridae* | 2171.76 |
| *Hipposideros armiger* | × | *Chiroptera* | *Hipposideridae* | 2236.58 |
| *Rhinolophus sinicus* | × | *Chiroptera* | *Rhinolophidae* | 2073.34 |
| *Rhinolophus ferrumequinum* | × | *Chiroptera* | *Rhinolophidae* | 2075.77 |
| *Cynopterus brachyotis* | × | *Chiroptera* | *Pteropodidae* | 1758.94 |
| *Cynopterus sphinx* | × | *Chiroptera* | *Pteropodidae* | 1857.07 |
| *Eonycteris spelaea* | × | *Chiroptera* | *Pteropodidae* | 1966.86 |
| *Macroglossus sobrinus* | × | *Chiroptera* | *Pteropodidae* | 1897.64 |
| *Pteropus pselaphon* | × | *Chiroptera* | *Pteropodidae* | 1933.22 |
| *Pteropus alecto* | × | *Chiroptera* | *Pteropodidae* | 1985.98 |
| *Pteropus giganteus* | × | *Chiroptera* | *Pteropodidae* | 1985.22 |
| *Pteropus vampyrus* | × | *Chiroptera* | *Pteropodidae* | 2198.28 |
| *Rousettus leschenaultii* | × | *Chiroptera* | *Pteropodidae* | 1918.04 |
| *Rousettus aegyptiacus* | × | *Chiroptera* | *Pteropodidae* | 1893.60 |
| *Arctocephalus gazella* | × | *Carnivora; Caniformia; Pinnipedia* | *Otariidae* | 2313.59 |
| *Arctocephalus townsendi* | × | *Carnivora; Caniformia; Pinnipedia* | *Otariidae* | 2372.37 |
| *Callorhinus ursinus* | × | *Carnivora; Caniformia; Pinnipedia* | *Otariidae* | 2706.87 |
| *Eumetopias jubatus* | × | *Carnivora; Caniformia; Pinnipedia* | *Otariidae* | 2418.26 |
| *Zalophus californianus* | × | *Carnivora; Caniformia; Pinnipedia* | *Otariidae* | 2409.69 |
| *Odobenus rosmarus divergens* | × | *Carnivora; Caniformia; Pinnipedia* | *Odobenidae* | 2400.15 |
| *Halichoerus grypus* | × | *Carnivora; Caniformia; Pinnipedia* | *Phocidae* | 2338.24 |
| *Leptonychotes weddellii* | × | *Carnivora; Caniformia; Pinnipedia* | *Phocidae* | 2455.28 |
| *Mirounga angustirostris* | × | *Carnivora; Caniformia; Pinnipedia* | *Phocidae* | 2366.17 |
| *Mirounga leonina* | × | *Carnivora; Caniformia; Pinnipedia* | *Phocidae* | 2417.32 |
| *Neomonachus schauinslandi* | × | *Carnivora; Caniformia; Pinnipedia* | *Phocidae* | 2419.44 |
| *Phoca vitulina* | × | *Carnivora; Caniformia; Pinnipedia* | *Phocidae* | 2363.59 |
| *Pusa hispida* | × | *Carnivora; Caniformia; Pinnipedia* | *Phocidae* | 2350.02 |
| *Eira barbara* | × | *Carnivora; Caniformia* | *Mustelidae* | 2469.72 |
| *Enhydra lutris* | × | *Carnivora; Caniformia* | *Mustelidae* | 2455.28 |
| *Gulo gulo* | × | *Carnivora; Caniformia* | *Mustelidae* | 2388.21 |
| *Lontra canadensis* | × | *Carnivora; Caniformia* | *Mustelidae* | 2405.75 |
| *Mustela lutreola* | × | *Carnivora; Caniformia* | *Mustelidae* | 2186.62 |
| *Mustela erminea* | × | *Carnivora; Caniformia* | *Mustelidae* | 2445.22 |
| *Mustela putorius furo* | × | *Carnivora; Caniformia* | *Mustelidae* | 2577.11 |
| *Taxidea taxus* | × | *Carnivora; Caniformia* | *Mustelidae* | 2416.01 |
| *Ailuropoda melanoleuca* | × | *Carnivora; Caniformia* | *Ursidae* | 2440.28 |
| *Tremarctos ornatus* | × | *Carnivora; Caniformia* | *Ursidae* | 2342.01 |
| *Ursus americanus* | × | *Carnivora; Caniformia* | *Ursidae* | 2588.39 |
| *Ursus thibetanus* | × | *Carnivora; Caniformia* | *Ursidae* | 2211.14 |
| *Ursus arctos* | × | *Carnivora; Caniformia* | *Ursidae* | 2474.26 |
| *Ursus maritimus* | × | *Carnivora; Caniformia* | *Ursidae* | 2330.49 |
| *Canis lupus familiaris* | × | *Carnivora; Caniformia* | *Canidae* | 2396.86 |
| *Lycaon pictus* | × | *Carnivora; Caniformia* | *Canidae* | 2137.70 |
| *Otocyon megalotis* | × | *Carnivora; Caniformia* | *Canidae* | 2374.66 |
| *Speothos venaticus* | × | *Carnivora; Caniformia* | *Canidae* | 2317.71 |
| *Vulpes ferrilata* | × | *Carnivora; Caniformia* | *Canidae* | 2379.66 |
| *Vulpes lagopus* | × | *Carnivora; Caniformia* | *Canidae* | 2345.55 |
| *Vulpes vulpes* | × | *Carnivora; Caniformia* | *Canidae* | 2421.57 |
| *Acinonyx jubatus* | × | *Carnivora; Feliformia* | *Felidae* | 2384.85 |
| *Felis catus* | × | *Carnivora; Feliformia* | *Felidae* | 2425.75 |
| *Lynx canadensis* | × | *Carnivora; Feliformia* | *Felidae* | 2407.81 |
| *Panthera pardus* | × | *Carnivora; Feliformia* | *Felidae* | 2578.02 |
| *Panthera tigris* | × | *Carnivora; Feliformia* | *Felidae* | 2408.70 |
| *Puma concolor* | × | *Carnivora; Feliformia* | *Felidae* | 2432.97 |
| *Puma yagouaroundi* | × | *Carnivora; Feliformia* | *Felidae* | 2473.14 |
| *Helogale parvula* | × | *Carnivora; Feliformia* | *Herpestidae* | 2392.47 |
| *Mungos mungo* | × | *Carnivora; Feliformia* | *Herpestidae* | 2448.21 |
| *Suricata suricatta* | × | *Carnivora; Feliformia* | *Herpestidae* | 2353.58 |
| *Crocuta crocuta* | × | *Carnivora; Feliformia* | *Hyaenidae* | 2348.38 |
| *Hyaena hyaena* | × | *Carnivora; Feliformia* | *Hyaenidae* | 2374.73 |
| *Proteles cristata* | × | *Carnivora; Feliformia* | *Hyaenidae* | 2388.97 |
| *Ceratotherium simum* | × | *Perissodactyla* | *Rhinocerotidae* | 2495.81 |
| *Ceratotherium simum simum* | × | *Perissodactyla* | *Rhinocerotidae* | 2870.08 |
| *Dicerorhinus sumatrensis* | × | *Perissodactyla* | *Rhinocerotidae* | 2442.45 |
| *Diceros bicornis* | × | *Perissodactyla* | *Rhinocerotidae* | 3005.52 |
| *Rhinoceros unicornis* | × | *Perissodactyla* | *Rhinocerotidae* | 2632.00 |
| *Equus asinus* | × | *Perissodactyla* | *Equidae* | 2431.91 |
| *Equus caballus* | × | *Perissodactyla* | *Equidae* | 2506.97 |
| *Equus przewalskii* | × | *Perissodactyla* | *Equidae* | 2395.95 |
| *Equus quagga* | × | *Perissodactyla* | *Equidae* | 2500.93 |
| *Camelus bactrianus* | × | *Artiodactyla; Tylopoda* | *Camelidae* | 1992.66 |
| *Camelus dromedarius* | × | *Artiodactyla; Tylopoda* | *Camelidae* | 2169.36 |
| *Camelus ferus* | × | *Artiodactyla; Tylopoda* | *Camelidae* | 2087.09 |
| *Lama glama* | × | *Artiodactyla; Tylopoda* | *Camelidae* | 2351.76 |
| *Lama guanicoe* | × | *Artiodactyla; Tylopoda* | *Camelidae* | 2050.53 |
| *Vicugna pacos* | × | *Artiodactyla; Tylopoda* | *Camelidae* | 2118.87 |
| *Vicugna vicugna* | × | *Artiodactyla; Tylopoda* | *Camelidae* | 2026.80 |
| *Phacochoerus africanus* | × | *Artiodactyla; Suina* | *Suidae* | 2435.08 |
| *Sus scrofa* | × | *Artiodactyla; Suina* | *Suidae* | 2501.91 |
| *Sus cebifrons* | × | *Artiodactyla; Suina* | *Suidae* | 2459.27 |
| *Moschus berezovskii* | × | *Artiodactyla; Ruminantia; Pecora* | *Moschidae* | 2813.37 |
| *Moschus chrysogaster* | × | *Artiodactyla; Ruminantia; Pecora* | *Moschidae* | 4972.48 |
| *Moschus moschiferus* | × | *Artiodactyla; Ruminantia; Pecora* | *Moschidae* | 3069.61 |
| *Cervus elaphus* | × | *Artiodactyla; Ruminantia; Pecora* | *Cervidae* | 2886.60 |
| *Cervus hanglu* | × | *Artiodactyla; Ruminantia; Pecora* | *Cervidae* | 2594.11 |
| *Muntiacus muntjak* | × | *Artiodactyla; Ruminantia; Pecora* | *Cervidae* | 2573.53 |
| *Muntiacus reevesi* | × | *Artiodactyla; Ruminantia; Pecora* | *Cervidae* | 2494.01 |
| *Odocoileus virginianus* | × | *Artiodactyla; Ruminantia; Pecora* | *Cervidae* | 2424.21 |
| *Giraffa camelopardalis* | × | *Artiodactyla; Ruminantia; Pecora* | *Giraffidae* | 2441.48 |
| *Giraffa tippelskirchi* | × | *Artiodactyla; Ruminantia; Pecora* | *Giraffidae* | 2490.08 |
| *Bison bison* | × | *Artiodactyla; Ruminantia; Pecora* | *Bovidae* | 2828.03 |
| *Bos indicus* | × | *Artiodactyla; Ruminantia; Pecora* | *Bovidae* | 2673.97 |
| *Bos indicus x Bos taurus* | × | *Artiodactyla; Ruminantia; Pecora* | *Bovidae* | 2680.95 |
| *Bos mutus* | × | *Artiodactyla; Ruminantia; Pecora* | *Bovidae* | 2645.16 |
| *Bos taurus* | × | *Artiodactyla; Ruminantia; Pecora* | *Bovidae* | 2711.21 |
| *Bubalus bubalis* | × | *Artiodactyla; Ruminantia; Pecora* | *Bovidae* | 2622.46 |
| *Oryx dammah* | × | *Artiodactyla; Ruminantia; Pecora* | *Bovidae* | 2707.18 |
| *Ovis aries* | × | *Artiodactyla; Ruminantia; Pecora* | *Bovidae* | 2628.15 |
