# Supplementary material for: Exploring objective feature sets in constructing the evolution relationship of animal genome sequences
Source: BMC Genomics. 2023 Oct 24;24:634. doi: 10.1186/s12864-023-09747-x (PMC10594854; doi:10.1186/s12864-023-09747-x)
Supplement: Supplementary file 2 — Additional file 2: Supplementary Table S2. The information of Reptilia species and genomes used in this study. [file 12864_2023_9747_MOESM2_ESM.docx]

**Supplementary table S2.** The information of *Reptilia* species and genomes used in this study.

| **Name** | **Taxonomic Category** | | **Genome**  **Size (Mb)** |
| --- | --- | --- | --- |
|  | **Order** | **Family** |  |
| *Chrysopelea ornata* | *Serpentiformes* | *Colubridae; Natricinae* | 1334.28 |
| *Pantherophis guttatus* | *Serpentiformes* | *Colubridae; Natricinae* | 1706.96 |
| *Pantherophis obsoletus* | *Serpentiformes* | *Colubridae; Natricinae* | 1692.46 |
| *Pituophis catenifer* | *Serpentiformes* | *Colubridae; Natricinae* | 1516.10 |
| *Ptyas mucosa* | *Serpentiformes* | *Colubridae; Natricinae* | 1721.47 |
| *Thamnophis elegans* | *Serpentiformes* | *Colubridae; Natricinae* | 1672.19 |
| *Thamnophis sirtalis* | *Serpentiformes* | *Colubridae; Natricinae* | 1424.90 |
| *Diadophis punctatus* | *Serpentiformes* | *Colubridae; Dipsadinae* | 1783.02 |
| *Erythrolamprus reginae* | *Serpentiformes* | *Colubridae; Dipsadinae* | 2038.23 |
| *Imantodes cenchoa* | *Serpentiformes* | *Colubridae; Dipsadinae* | 1385.16 |
| *Thermophis baileyi* | *Serpentiformes* | *Colubridae; Dipsadinae* | 1747.68 |
| *Bothrops jararaca* | *Serpentiformes* | *Viperidae* | 1672.32 |
| *Crotalus adamanteus* | *Serpentiformes* | *Viperidae* | 1597.65 |
| *Crotalus horridus* | *Serpentiformes* | *Viperidae* | 1520.33 |
| *Crotalus pyrrhus* | *Serpentiformes* | *Viperidae* | 1126.79 |
| *Crotalus tigris* | *Serpentiformes* | *Viperidae* | 1612.00 |
| *Crotalus viridis* | *Serpentiformes* | *Viperidae* | 1340.20 |
| *Protobothrops flavoviridis* | *Serpentiformes* | *Viperidae* | 1413.20 |
| *Protobothrops mucrosquamatus* | *Serpentiformes* | *Viperidae* | 1673.88 |
| *Vipera berus* | *Serpentiformes* | *Viperidae* | 1532.39 |
| *Emydocephalus ijimae* | *Serpentiformes* | *Elapidae* | 1625.20 |
| *Hydrophis melanocephalus* | *Serpentiformes* | *Elapidae* | 1402.64 |
| *Hydrophis curtus* | *Serpentiformes* | *Elapidae* | 1964.83 |
| *Hydrophis cyanocinctus* | *Serpentiformes* | *Elapidae* | 1980.71 |
| *Hydrophis hardwickii* | *Serpentiformes* | *Elapidae* | 1296.39 |
| *Laticauda colubrina* | *Serpentiformes* | *Elapidae* | 2038.82 |
| *Laticauda laticaudata* | *Serpentiformes* | *Elapidae* | 1558.71 |
| *Naja naja* | *Serpentiformes* | *Elapidae* | 1768.54 |
| *Notechis scutatus* | *Serpentiformes* | *Elapidae* | 1665.53 |
| *Pseudonaja textilis* | *Serpentiformes* | *Elapidae* | 1590.04 |
| *Actinemys pallida* | *Testudines* | *Testudinoidea; Emydidae* | 2326.93 |
| *Actinemys marmorata* | *Testudines* | *Testudinoidea; Emydidae* | 2301.12 |
| *Chrysemys picta* | *Testudines* | *Testudinoidea; Emydidae* | 2481.37 |
| *Emys orbicularis* | *Testudines* | *Testudinoidea; Emydidae* | 2309.74 |
| *Malaclemys terrapin* | *Testudines* | *Testudinoidea; Emydidae* | 2439.75 |
| *Terrapene carolina* | *Testudines* | *Testudinoidea; Emydidae* | 2571.27 |
| *Trachemys scripta* | *Testudines* | *Testudinoidea; Emydidae* | 2126.20 |
| *Platysternon megacephalum* | *Testudines* | *Testudinoidea; Emydidae* | 2319.09 |
| *Cuora amboinensis* | *Testudines* | *Testudinoidea; Geoemydidae* | 2214.83 |
| *Cuora mccordi* | *Testudines* | *Testudinoidea; Geoemydidae* | 2390.37 |
| *Mauremys mutica* | *Testudines* | *Testudinoidea; Geoemydidae* | 2484.37 |
| *Mauremys reevesii* | *Testudines* | *Testudinoidea; Geoemydidae* | 2367.61 |
| *Aldabrachelys gigantea* | *Testudines* | *Testudinoidea; Testudinidae* | 2373.37 |
| *Chelonoidis abingdonii* | *Testudines* | *Testudinoidea; Testudinidae* | 2300.74 |
| *Gopherus agassizii* | *Testudines* | *Testudinoidea; Testudinidae* | 2184.97 |
| *Gopherus evgoodei* | *Testudines* | *Testudinoidea; Testudinidae* | 2298.56 |
| *Gopherus flavomarginatus* | *Testudines* | *Testudinoidea; Testudinidae* | 2460.54 |
| *Caretta caretta* | *Testudines* | *Chelonioidea; Cheloniidae* | 2134.01 |
| *Chelonia mydas* | *Testudines* | *Chelonioidea; Cheloniidae* | 2134.38 |
| *Eretmochelys imbricata* | *Testudines* | *Chelonioidea; Cheloniidae* | 2296.23 |
| *Dermochelys coriacea* | *Testudines* | *Chelonioidea; Dermochelyidae* | 2164.76 |
| *Emydura macquarii* | *Testudines* | *Chelidae* | 1924.52 |
| *Emydura subglobosa* | *Testudines* | *Chelidae* | 1986.70 |
| *Mesoclemmys tuberculata* | *Testudines* | *Chelidae* | 2027.51 |
| *Apalone spinifera* | *Testudines* | *Trionychia; Trionychidae* | 1931.08 |
| *Pelodiscus sinensis* | *Testudines* | *Trionychia; Trionychidae* | 2202.48 |
| *Rafetus swinhoei* | *Testudines* | *Trionychia; Trionychidae* | 2237.95 |
| *Carettochelys insculpta* | *Testudines* | *Trionychia; Carettochelyidae* | 2356.57 |
