# Supplementary material for: Exploring objective feature sets in constructing the evolution relationship of animal genome sequences
Source: BMC Genomics. 2023 Oct 24;24:634. doi: 10.1186/s12864-023-09747-x (PMC10594854; doi:10.1186/s12864-023-09747-x)
Supplement: Supplementary file 3 — Additional file 3: Supplementary Table S3. The information of Insecta species and genomes used in this study. [file 12864_2023_9747_MOESM3_ESM.docx]

**Supplementary table S3.** The information of *Insecta* species and genomes used in this study.

| **Name** | **Taxonomic Category** | | **Genome Size (Mb)** |
| --- | --- | --- | --- |
|  | **Order** | **Family** |  |
| *Anopheles albimanus* | *Diptera* | *Culicidae* | 172.603 |
| *Anopheles arabiensis* | *Diptera* | *Culicidae* | 256.823 |
| *Anopheles coluzzii* | *Diptera* | *Culicidae* | 273.424 |
| *Anopheles gambiae* | *Diptera* | *Culicidae* | 265.027 |
| *Anopheles stephensi* | *Diptera* | *Culicidae* | 243.460 |
| *Culex pipiens* | *Diptera* | *Culicidae* | 566.674 |
| *Culex quinquefasciatus* | *Diptera* | *Culicidae* | 573.230 |
| *Drosophila albomicans* | *Diptera* | *Drosophilidae* | 165.846 |
| *Drosophila arizonae* | *Diptera* | *Drosophilidae* | 141.387 |
| *Drosophila bipectinata* | *Diptera* | *Drosophilidae* | 192.394 |
| *Drosophila elegans* | *Diptera* | *Drosophilidae* | 178.445 |
| *Drosophila ficusphila* | *Diptera* | *Drosophilidae* | 167.833 |
| *Drosophila guanche* | *Diptera* | *Drosophilidae* | 140.633 |
| *Drosophila innubila* | *Diptera* | *Drosophilidae* | 167.978 |
| *Drosophila melanogaster* | *Diptera* | *Drosophilidae* | 143.726 |
| *Drosophila miranda* | *Diptera* | *Drosophilidae* | 287.096 |
| *Drosophila mojavensis* | *Diptera* | *Drosophilidae* | 178.498 |
| *Drosophila navojoa* | *Diptera* | *Drosophilidae* | 147.358 |
| *Drosophila novamexicana* | *Diptera* | *Drosophilidae* | 177.224 |
| *Drosophila persimilis* | *Diptera* | *Drosophilidae* | 195.513 |
| *Drosophila rhopaloa* | *Diptera* | *Drosophilidae* | 193.508 |
| *Drosophila sechellia* | *Diptera* | *Drosophilidae* | 153.085 |
| *Drosophila takahashii* | *Diptera* | *Drosophilidae* | 165.528 |
| *Drosophila yakuba* | *Diptera* | *Drosophilidae* | 147.899 |
| *Bactrocera dorsalis* | *Diptera* | *Tephritidae* | 414.985 |
| *Bactrocera latifrons* | *Diptera* | *Tephritidae* | 462.505 |
| *Bactrocera oleae* | *Diptera* | *Tephritidae* | 409.659 |
| *Bactrocera tryoni* | *Diptera* | *Tephritidae* | 570.659 |
| *Ceratitis capitata* | *Diptera* | *Tephritidae* | 436.491 |
| *Rhagoletis pomonella* | *Diptera* | *Tephritidae* | 1223.29 |
| *Rhagoletis zephyria* | *Diptera* | *Tephritidae* | 1109.80 |
| *Zeugodacus cucurbitae* | *Diptera* | *Tephritidae* | 374.820 |
| *Acyrthosiphon pisum* | *Hemiptera* | *Aphididae* | 533.649 |
| *Aphis gossypii* | *Hemiptera* | *Aphididae* | 294.279 |
| *Diuraphis noxia* | *Hemiptera* | *Aphididae* | 395.074 |
| *Melanaphis sacchari* | *Hemiptera* | *Aphididae* | 300.272 |
| *Myzus persicae* | *Hemiptera* | *Aphididae* | 347.313 |
| *Rhopalosiphum maidis* | *Hemiptera* | *Aphididae* | 326.023 |
| *Sipha flava* | *Hemiptera* | *Aphididae* | 353.188 |
| *Apis cerana* | *Hymenoptera* | *Apidae* | 221.320 |
| *Apis dorsata* | *Hymenoptera* | *Apidae* | 230.340 |
| *Apis florea* | *Hymenoptera* | *Apidae* | 229.015 |
| *Apis mellifera* | *Hymenoptera* | *Apidae* | 225.251 |
| *Bombus bifarius* | *Hymenoptera* | *Apidae* | 266.785 |
| *Bombus impatiens* | *Hymenoptera* | *Apidae* | 246.856 |
| *Bombus terrestris* | *Hymenoptera* | *Apidae* | 392.962 |
| *Bombus vancouverensis* | *Hymenoptera* | *Apidae* | 282.134 |
| *Bombus vosnesenskii* | *Hymenoptera* | *Apidae* | 275.578 |
| *Aphidius gifuensis* | *Hymenoptera* | *Braconidae* | 156.965 |
| *Chelonus insularis* | *Hymenoptera* | *Braconidae* | 135.730 |
| *Diachasma alloeum* | *Hymenoptera* | *Braconidae* | 384.372 |
| *Fopius arisanus* | *Hymenoptera* | *Braconidae* | 153.632 |
| *Microplitis demolitor* | *Hymenoptera* | *Braconidae* | 241.190 |
| *Harpegnathos saltator* | *Hymenoptera* | *Formicidae* | 334.162 |
| *Linepithema humile* | *Hymenoptera* | *Formicidae* | 219.501 |
| *Monomorium pharaonis* | *Hymenoptera* | *Formicidae* | 325.507 |
| *Nylanderia fulva* | *Hymenoptera* | *Formicidae* | 369.948 |
| *Odontomachus brunneus* | *Hymenoptera* | *Formicidae* | 393.037 |
| *Ooceraea biroi* | *Hymenoptera* | *Formicidae* | 223.876 |
| *Solenopsis invicta* | *Hymenoptera* | *Formicidae* | 378.102 |
| *Temnothorax curvispinosus* | *Hymenoptera* | *Formicidae* | 303.539 |
| *Trachymyrmex cornetzi* | *Hymenoptera* | *Formicidae* | 369.079 |
| *Trachymyrmex septentrionalis* | *Hymenoptera* | *Formicidae* | 291.747 |
| *Trachymyrmex zeteki* | *Hymenoptera* | *Formicidae* | 267.973 |
| *Ostrinia furnacalis* | *Lepidoptera* | *Crambidae* | 437.294 |
| *Helicoverpa armigera* | *Lepidoptera* | *Noctuidae* | 337.088 |
| *Spodoptera frugiperda* | *Lepidoptera* | *Noctuidae* | 485.970 |
| *Spodoptera litura* | *Lepidoptera* | *Noctuidae* | 429.583 |
| *Trichoplusia ni* | *Lepidoptera* | *Noctuidae* | 368.211 |
| *Bicyclus anynana* | *Lepidoptera* | *Nymphalidae* | 475.400 |
| *Danaus plexippus* | *Lepidoptera* | *Nymphalidae* | 248.676 |
| *Maniola hyperantus* | *Lepidoptera* | *Nymphalidae* | 408.137 |
| *Pararge aegeria* | *Lepidoptera* | *Nymphalidae* | 516.571 |
| *Vanessa tameamea* | *Lepidoptera* | *Nymphalidae* | 357.125 |
| *Papilio machaon* | *Lepidoptera* | *Papilionidae* | 252.114 |
| *Papilio polytes* | *Lepidoptera* | *Papilionidae* | 227.021 |
| *Papilio xuthus* | *Lepidoptera* | *Papilionidae* | 243.890 |
| *Amyelois transitella* | *Lepidoptera* | *Pyralidae* | 406.468 |
| *Galleria mellonella* | *Lepidoptera* | *Pyralidae* | 401.915 |
